# Effects of Thermally-Oxidized Frying Oils (Corn Oil and Lard) on Gut Microbiota in Hamsters

**DOI:** 10.3390/antiox11091732

**Published:** 2022-08-31

**Authors:** Erika Kwek, Chi Yan, Huafang Ding, Wangjun Hao, Zouyan He, Ka Ying Ma, Jianhui Liu, Hanyue Zhu, Zhen-Yu Chen

**Affiliations:** 1 School of Life Sciences, The Chinese University of Hong Kong, Shatin, Hong Kong 999077, China; 2 School of Public Health, Guangxi Medical University, Nanning 530021, China; 3 College of Food Science and Engineering, Nanjing University of Finance & Economics, Nanjing 210023, China; 4 School of Food Science and Engineering, South China Food Safety Research Center, Foshan University, Foshan 528200, Guangdong, China

**Keywords:** lipid oxidation, oxidized oil, gut microbiota, frying oils, lipid metabolism, oxidation, food

## Abstract

Repeated reuse of frying oil raises health concerns due to the accumulation of oxidative products after each frying cycle. Gut microbiota is integral in lipid metabolism and immune regulation. The present study was designed to investigate the effects of thermally-oxidized corn oil and lard on gut microbiota in relation to atherosclerosis, inflammatory cytokines, and plasma lipids. Male Golden Syrian hamsters were randomly divided into four groups and fed one of four diets containing fresh corn oil (CF), oxidized corn oil (CO), fresh lard (LF), and oxidized lard (LO), for six weeks. CO and LO were prepared by deep-frying potatoes in corn oil or lard for seven days. Results indicated that oxidized oil and lard caused the loss of species diversity and richness of gut microbiota. Feeding CO and LO also reduced the body and adipose tissue weights, associated with genus *Acetatifactor* and *Allobaculum*. Plasma triacylglycerols significantly increased by 51% in the CO and 35% in the LO group compared with that in their CF and LF counterparts, respectively. CO could also affect the abundance of specific bacteria genera: *Bacteroides, Barnesiella, Acetatifactor, Allobaculum, Clostridium_IV, Clostridium_XIVa, Coprococcus, Lactococcus, Paraprevotella, Parasutterella,* and *Oscillibacter*. In addition, CO and LO could adversely remodel gut composition and affect intestinal production of short-chain fatty acids, pro-inflammatory biomarkers (LPS and IL-6), anti-inflammatory biomarker IL-10, and atherosclerotic progression. It was concluded that frying oil could adversely modulate the gut microbiota and exacerbate the atherosclerosis at least in a hypercholesterolemia hamster model.

## 1. Introduction

The trend of fast-food consumption is reportedly increasing world-wide. One of the most popular fast-foods are French fries, prepared by deep-frying potato (*Solanum tuberosum*) [1]. Deep-frying is a process where food is submerged in hot oil and undergoes complex chemical reactions [2]. Loss of water from food can trigger the hydrolysis and breakage of triacylglycerol (TG) ester linkages, and releases the free fatty acids (FFA), glycerol, and mono-/di-acylglycerols [3]. Other chemical changes include the oxidation and polymerization of oil. Nonvolatile polar compounds, such as dimers and oligomers, are products of oil degradation [4]. FFA, volatile, and nonvolatile compounds alter the stability and quality of frying oils [5]. Deep-fat frying leads to a loss of unsaturated fatty acids and the physical increase in viscosity, color pigmentation, and foaming of oil [3,6]. Frying oils can be categorized based on their origin: vegetable oils or animal fats.

Corn is the most abundantly produced crop around the world with approximately 1000 million tons of annual yield, accounting for more than 30% total cereal production [7]. Corn oil is a vegetable oil that can be extracted from the corn germ either mechanically through expeller pressing, chemically using solvents like hexane, or a combination of these methods [8]. The typical fatty acid composition of corn oil is characterized by possessing a high amount of unsaturated fatty acid—particularly linoleic and oleic acid [8,9]. Corn oil is often used for cooking and deep-frying [10]. As one of the most common edible animal fats, lard is also used in the production of margarines, shortenings, sausages, and in deep-frying [11,12,13,14]. Compared with corn oil, lard contains more palmitic and stearic acids and less linoleic acid [12].

The repeated reuse of frying oil is a common practice in many fast-food restaurants. This has caused health concerns due to the accumulation of oxidative products after each frying cycle. To date, there has been no study to directly investigate the association of the intake of thermally-abused frying oils with diseases such as cardiovascular disease (CVD) [13,14]. It is known that diets can significantly modulate the gut microbiota. However, it remains unclear how frying oils affect the gut microbiota. Hence, the present study aims to investigate and compare the effects of thermally-abused corn oil (as a representative of vegetable oils) and lard (as a representative of animal fats) on gut microbiota in hamsters.

## 2. Materials and Methods

### 2.1. Oil Preparation

Corn oil (Mazola), lard (Camelia-Holland), and potatoes were purchased from the local market (New Territories, Hong Kong SAR, China). After the removal of skin, the potatoes were rinsed in cold water and cut into traditional 1.5-inch-thick French fries. Corn oil or lard (~2.5 L or 2200 g) was poured into the inner bowl of a Philips Cucina fryer (HD6155). Approximately 500 g of French fries were fried at 160 °C for 4 min, during the first frying cycle. Then, the potatoes were taken out and cooled at room temperature. The same batch of potatoes were submerged in 180 °C for 8 min during the second frying cycle. This two-step frying was repeated for 12 consecutive hours daily, using a fresh batch of potatoes each time. At the end of the 12 h of frying, the corn oil or lard was stored at room temperature overnight. The entire procedure was continued for 7 days for both corn oil and lard to produce oxidized corn oil and oxidized lard, respectively.

### 2.2. Diet Preparation

Four diets were formulated based on the American Institute of Nutrition (AIN)-93 animal diet, with minor adjustments [15,16] (Table 1). The basal diets were made with (g/kg diet): corn starch (508), casein (242), sucrose (19), mineral mix (40), vitamin mix (20), gelatin (20), DL-methionine (1), and cholesterol (0.4). Each diet consisted of 30% kcal fat by adding 155g/kg of either fresh corn oil (CF), oxidized corn oil (CO), fresh lard (LF), or oxidized lard (LO). All four diets were molded, air-dried, and stored at −20 °C.

### 2.3. Peroxide Value of Oil and Fats

The peroxide values of corn oil (fresh and oxidized), and lard (fresh and oxidized) were determined according to the AOAC Official Method 965.33. In brief, approximately 5 g of oils or fats was weighed into a conical flask. A mixture of 30 mL glacial acetic acid–chloroform (3:2) was added. Addition of 0.5 mL saturated potassium iodide was followed by 30 mL of ddH_2_O. After mixing, 0.5 mL of 1% starch solution was added. Titration was slowly conducted using 0.01 M sodium thiosulfate (Na_2_S_2_O_3_) until reversed color change was observed. The final and initial volume of Na_2_S_2_O_3_ were recorded. Peroxide values (mEq peroxide/kg sample) were calculated according to the formula:

(1) $$Peroxide\;value=\frac{Volume\;of\;{Na}_{2}S_{2}O_{3}\;used\;\times \;Molarity\;\mathrm{of}\;{Na}_{2}S_{2}O_{3}\;\times \;1000}{Sample\;weight\;(g)}$$

### 2.4. Fatty Acid Analyses

The fatty acid compositions of all the corn oil and lard were analyzed using a gas liquid chromatography method as described previously [17]. Boron trifluoride (14% in methanol) was added to corn oils and lards for methylation and the preparation of fatty acid methyl esters (FAMEs). A Shimadzu Gas Chromatograph 2010 (Shimadzu, Tokyo, Japan) with an HP Innowax column (Agilent Technologies, Santa Clara, CA, USA) was used to separately detect individual FAMEs according to retention time compared to standards (Nu-Chek Prep Inc., Elysian, MN, USA). Quantification was conducted relative to the internal standard of heptadecanoic acid.

### 2.5. Animals

Male, eight-week-old Golden Syrian hamsters (*Mesocricetus auratus*) were selected as an animal model. The entire experimental protocol was approved by the Animal Experimental Ethical Committee of the Chinese University of Hong Kong (Reference Number: 21-169-MIS). Hamsters with 100–120 g body weight were randomly divided into four groups (*n* = 8, each). The animal housing room was equipped with controlled environmental settings (12 h–12 h light–dark cycle, 23 °C room temperature and free access to tap water). After one week of acclimatization, the hamsters were fed one of four diets (CF, CO, LF, LO) for six weeks (Table 1). Body weight, food intake, and fecal outputs were recorded weekly. Hamsters were sacrificed using carbon dioxide anesthesia after six weeks’ feeding. Blood, organ, and tissue collections were conducted similarly to the procedures described above [17].

### 2.6. Plasma Lipid Profile Analysis

Plasma lipoprotein and lipids were quantified including total cholesterol (TC), triacylglycerols (TG), high-density lipoprotein cholesterol (HDL-C), and non-high-density lipoprotein cholesterol (n-HDL-C). The plasma samples of the hamsters were tested at Week 0 and week 6 using analytic kits (Stanbio Laboratories, Boerne, TX, USA). The value of n-HDL-C was calculated by subtracting HDL-C from TC.

### 2.7. Atherosclerotic Plaque Quantification

Atherosclerosis was measured by staining the thoracic aorta with Oil Red O. The staining preparation involved prior cleaning and removal of tissues around the aorta. Individual aorta were cut vertically, and dried by isopropanol. After staining, the aorta was scanned by desktop scanner and quantified through ImageJ software (NIH, LOCI, USA), to measure the plaque area percentage.

### 2.8. Analysis of Plasma LPS, IL-10, and IL-6

Plasma lipopolysaccharide (LPS), interleukin 10 (IL-10), and IL-6 were measured using enzyme-linked immunosorbent assay (ELISA) kits (Cusabio Technology, Wuhan, Hubei, China). Briefly, the standards provided were diluted serially to create a standard curve. Plasma samples from Week 6 were added to pre-coated antibody wells. After the addition of reagents, absorbances were measured using the spectrophotometer.

### 2.9. Analysis of Fecal Short-Chain Fatty Acids (SCFA)

Total SCFA includes acetic acid, propionic acid, butyric acid, and valeric acid. Fecal total SCFA was quantified through methods previously described [18,19]. The feces collected at Week 6 were extracted using 50% ethanol. Internal standard 2-ethylbutyric acid was also added. After repeated sonication and centrifugation, the supernatant was injected into a gas chromatograph (Shimadzu GC-2010, Tokyo, Japan) with CP-FFAP CB capillary column (Agilent Technologies, CA, USA), and quantified according to the amount of internal standard added.

### 2.10. 16S rRNA Gene Sequencing

Fresh feces collected at Week 6 were used for 16S rRNA gene sequencing of gut microbiota (BGI Genomics, New Territories, Hong Kong SAR, China). Bacterial DNA templates were amplified with 16S fusion primers targeting V3-V4 regions. After PCR, purification, and library construction, sequencing was conducted using Illumina HiSeq2500. Clustering into operational taxonomic units (OTUs) was conducted using USEARCH (version 7.0.1090), where tags have a 97% similarity threshold. Annotation of taxonomy based on OTUs was conducted through Greengene database by RDP classifier (version 2.2), and analyses were conducted using R software (version 3.1.1).

### 2.11. Statistical Analysis

Data were presented as mean ± SD. One-way ANOVA and LSD post-hoc test was used to detect the statistical differences among the four diet groups when *p* < 0.05 (SPSS Inc., version 15.0, IBM, Bloomington, IL, USA). Gut microbiota comparisons between four different groups were conducted using the Kruskal–Wallis test. The relationship between bacterial abundance and environmental parameters was evaluated using Spearman’s correlation.

## 3. Results

### 3.1. Fatty Acid Profile and Peroxide Value of Oil and Fats

Fresh corn oil was composed of 53% polyunsaturated fatty acids (primarily 18:2n-6) and 30% oleic acid (18:1n-9). Comparatively, fresh lard mainly consisted of 45% saturated fatty acids and 37% oleic acid. The deep-frying preparation process of oxidized corn oil and oxidized lard caused a 36% and 89% loss of polyunsaturated fatty acids, respectively (Table 2). Peroxide value is a widely accepted measure of hydroperoxide oxidation products to indicate oil or fat rancidity. Oxidized lard and corn oil had markedly elevated peroxide values after repeated deep-frying, compared to fresh lard and corn oil, respectively (Table 2).

### 3.2. Food Intake and Weights of Body, Organ, and Fecal Output

Hamsters across the five diet groups had similar daily food intakes. At the start of week 0, body weights among the groups were comparable. However, after 6 weeks of feeding, the CO and LO groups had 15% and 7% lower body weights (*p* < 0.05) compared with the CF and LF groups, respectively. Perirenal and epididymal adipose tissues are the major internal fat reserves of hamsters. The weights of both perirenal and epididymal fats were significantly decreased (*p* < 0.05) after intake of oxidized oil and lard. It was also observed that the total weights of feces excreted in week 6 by oxidized groups CO and LO were significantly elevated, by 93–111% (*p* < 0.05), compared with their CF and LF counterparts (Table 3).

### 3.3. Plasma Lipid Profiles

At week 0, the plasma lipid profiles of all the hamsters were statistically similar (*p* > 0.05). After six weeks’ feeding with oxidized lard, the LO group had a 13% (*p* < 0.05) lower plasma TC compared to LF. TC levels between the CF and CO groups remained comparable (*p* > 0.05). The plasma TG level in hamsters fed CO was 51% higher than the CF group; the LO group also had 35% more TG in plasma than the LF group (*p* < 0.05) (Table 4).

### 3.4. Atherosclerotic Plaque Area

**Figure 1:**
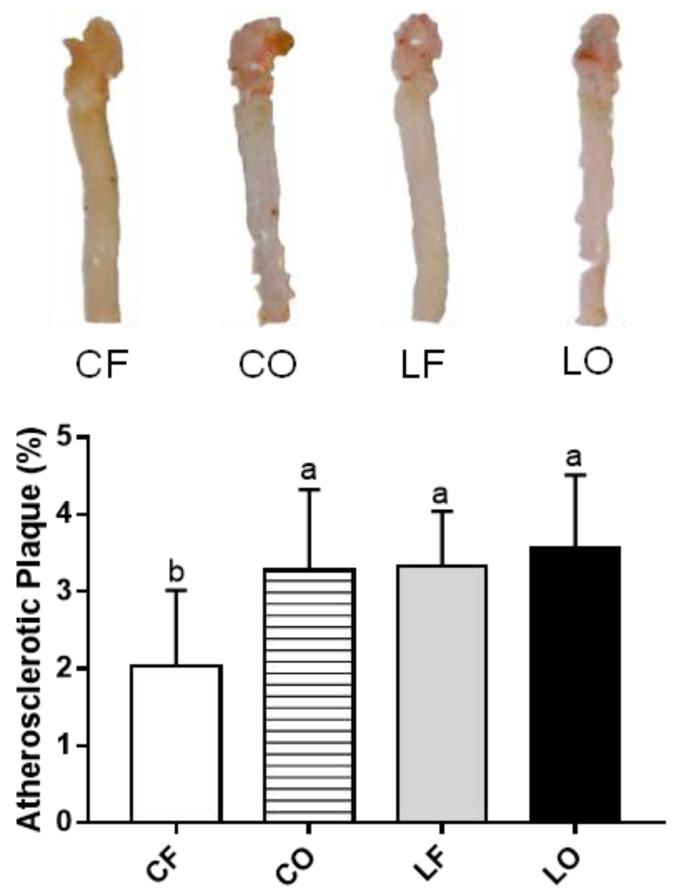
Atherosclerosis plaque areas in hamsters fed one of the four diets: CF, fresh corn oil; CO, oxidized corn oil; LF, fresh lard; LO, oxidized lard. Data are expressed as mean ± S.D., where different letters indicate statistical difference, *p* < 0.05.

Feeding a CO diet increased the atherosclerotic plaque in the aorta by 62% compared with feeding CF (*p* < 0.05). Plaque accumulation of the LF and LO groups was statistically similar (*p* > 0.05) (Figure 1).

### 3.5. Plasma LPS, IL-10, and IL-6

**Figure 2:**
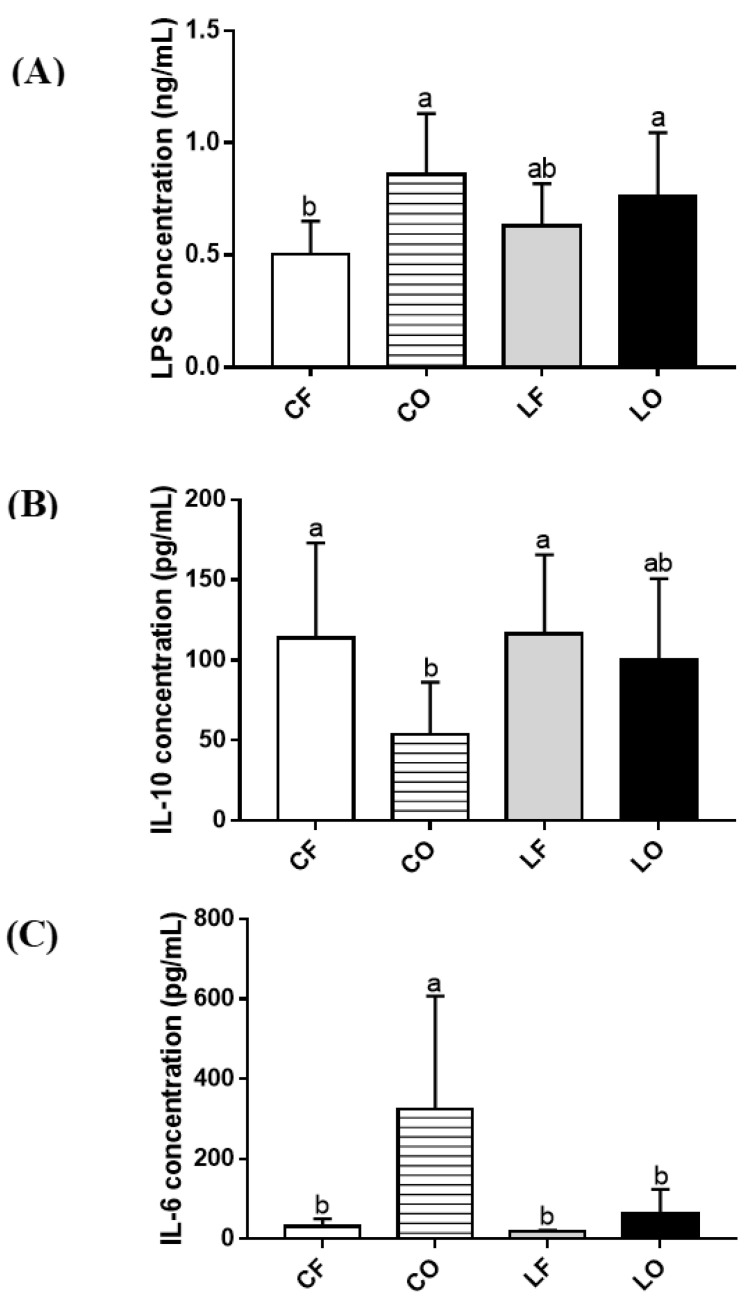
Concentration of (**A**) LPS; (**B**) IL-10; and (**C**) IL-6, in plasma of hamsters fed one of the four diets: CF, fresh corn oil; CO, oxidized corn oil; LF, fresh lard; LO, oxidized lard. Data are expressed as mean ± S.D., where different letters indicate statistical difference, *p* < 0.05.

Plasma concentrations of inflammatory biomarker LPS and IL-6 and anti-inflammatory IL-10 between the two lard groups (LF and LO) were relatively similar (*p* > 0.05). In contrast, compared to CF, the intake of CO dramatically increased plasma LPS by 72%, elevated IL-6 by 960%, and reduced IL-10 by 53% (*p* < 0.05) (Figure 2).

### 3.6. Fecal SCFA

**Figure 3:**
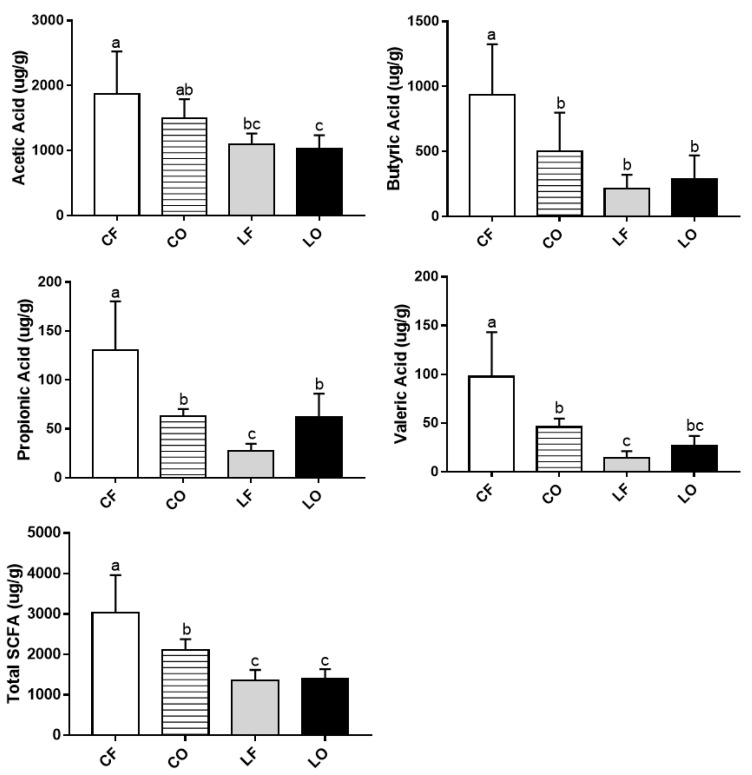
Changes in propionic acid, acetic acid, butyric acid, valeric acid, and total short chain fatty acids (SCFA) in hamsters fed one of the four diets: CF, fresh corn oil; CO, oxidized corn oil; LF, fresh lard; LO, oxidized lard. Data are expressed as mean ± S.D., where different letters indicate statistical difference, *p* < 0.05.

Total SCFA is the sum of acetic acid, propionic acid, butyric acid, and valeric acid. Consumption of CO led to a significant decrease in total fecal SCFA by 30% compared to CF. This is mainly characterized by the reduction of fecal propionic acid by 52%, butyric acid by 46%, and valeric acid by 53% (*p* < 0.05). Total fecal SCFA of LO and LF were statistically similar (*p* > 0.05) (Figure 3).

### 3.7. Operational Taxonomic Units (OTUs)

**Figure 4:**
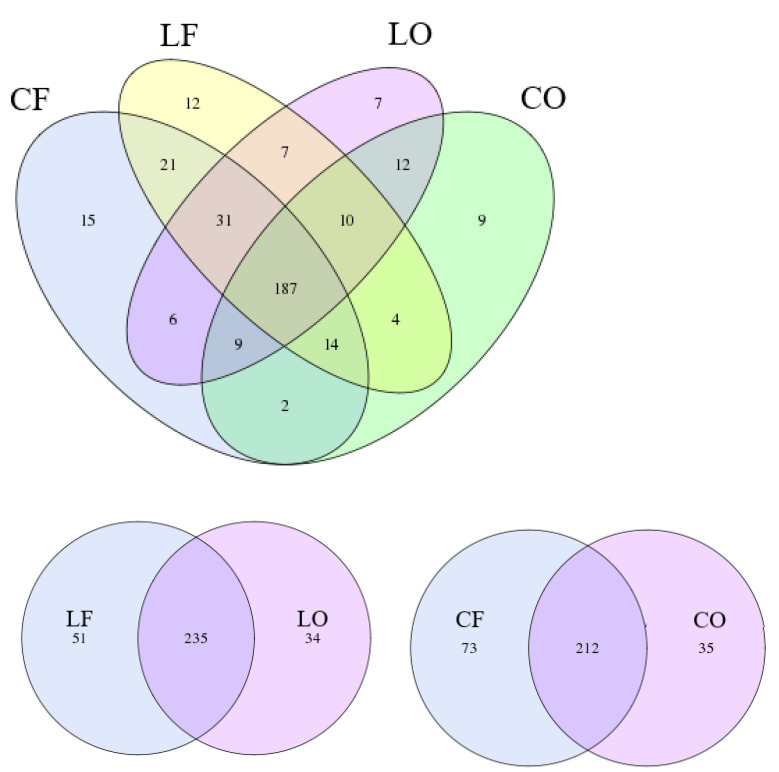
Venn diagrams representing unique and shared operational taxonomic units (OTUs) in hamsters fed one of the four diets: CF, fresh corn oil; CO, oxidized corn oil; LF, fresh lard; LO, oxidized lard.

**Figure 5:**
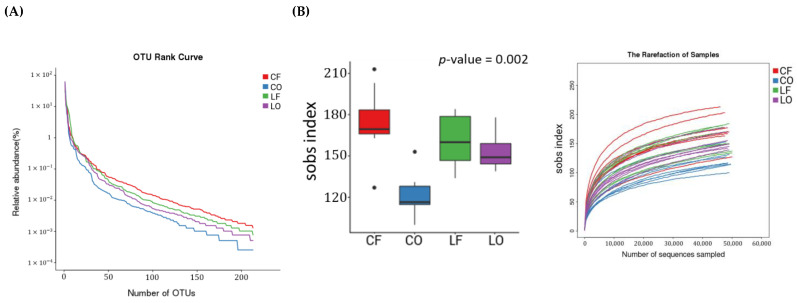

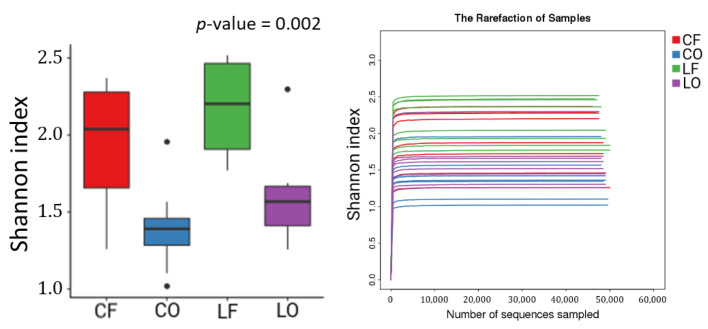
(**A**) Species diversity depicted by an Operational taxonomic units (OTU) rank curve; (**B**) Rarefaction curves and alpha diversity indices, comparing community richness (sobs) and diversity (Shannon) between hamsters fed one of the four diets: CF, fresh corn oil; CO, oxidized corn oil; LF, fresh lard; LO, oxidized lard.

Operational taxonomic units (OTUs) are clusters of bacteria that exhibit ≥97% similarity in 16S rRNA sequences. Venn diagrams represent the comparison of the OTUs of CF, CO, LF, and LO. It was observed that there were 187 shared OTUs between all four groups. Between same fat types: CF and CO shared 212 OTUs, while LF and LO shared 235 OTUs. In general, the fresh groups CF and LF had a higher number of unique OTUs compared to the oxidized groups CO and LO, respectively (Figure 4). An OTU rank curve is constructed by calculating the relative abundance of individual OTUs in each sample and ranking them in a decreasing order. A flatter curve shape indicates greater species evenness in a group. Among all four diet groups, species evenness and richness ranked in a decreasing order was: CF > LF > LO > CO. (Figure 5A).

### 3.8. Alpha Diversity

The alpha diversity index aims to measure sample community diversity. The Sobs (observed species) index reflects species richness, while the Shannon index represents species diversity. The shape of the alpha diversity rank curves (Sobs and Shannon) plateaued smoothly, indicating that sufficient samplings were conducted. In a decreasing order, the Sobs index of species richness between the groups was: CF > LF > LO > CO. The Shannon index of species diversity was: LF > CF > LO > CO. Alpha diversity analyses indicated that oxidized oils and fats decreased the richness and diversity of bacterial communities (*p* < 0.05) (Figure 5B).

### 3.9. Principal Component Analysis (PCA)

**Figure 6:**
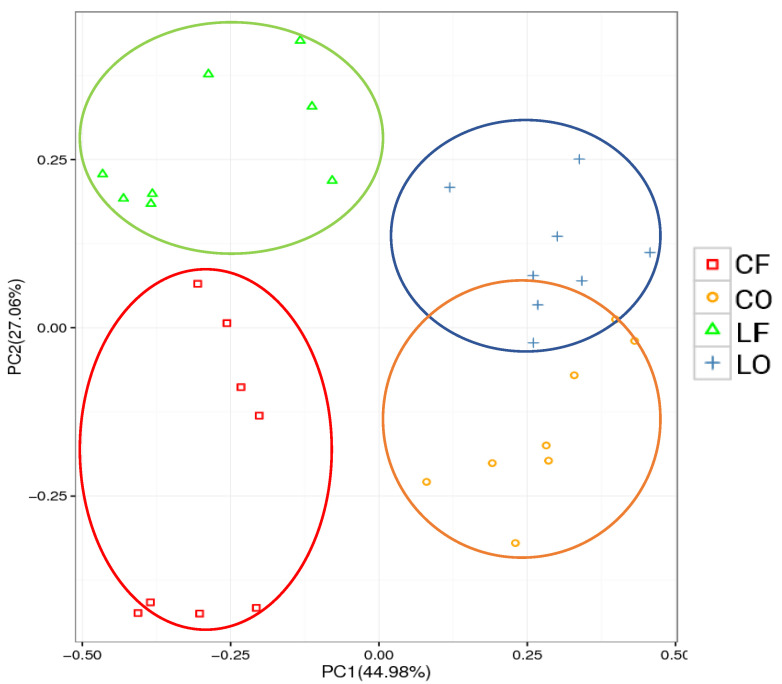
Principal components analysis (PCA) on operational taxonomic units (OTU) level, representing overall composition similarity and dissimilarity among groups of hamsters fed one of the four diets: CF, fresh corn oil; CO, oxidized corn oil; LF, fresh lard; LO, oxidized lard.

To visualize similarity or dissimilarity among groups, the dimensional reduction of PCA was conducted. On the OTU level, the composition clusters of oxidized groups and fresh groups were separated distinctively. It was observed that the CO and LO group clusters migrated along the PC1 axis, away from their respective CF and LF groups. PCA analysis indicated that oxidized fats and oils had a dissimilar bacteria composition compared with their corresponding non-oxidized counterparts (Figure 6).

### 3.10. Phyla Abundance

**Figure 7:**
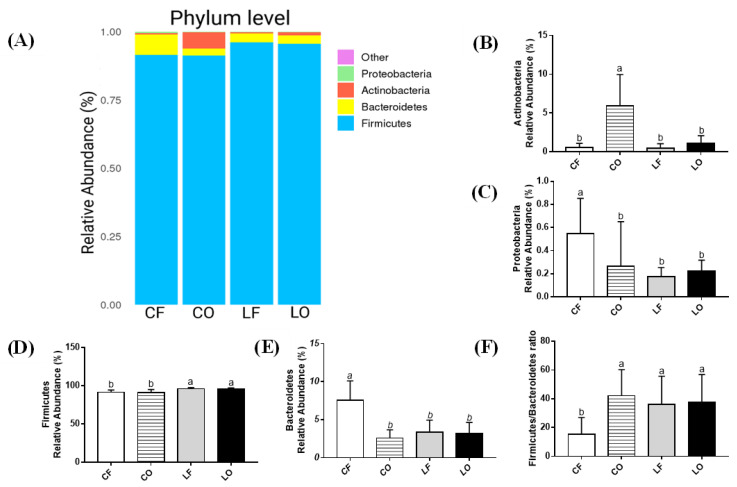
Phylum level changes in bacterial composition (>0.01%) of hamsters fed one of the four diets: CF, fresh corn oil; CO, oxidized corn oil; LF, fresh lard; LO, oxidized lard. (**A**) Bar plot of relative abundance (%). Comparison in abundances of: (**B**) Actinobacteria, (**C**) Proteobacteria, (**D**) Firmicutes, and (**E**) Bacteroidetes. (**F**) Ratio between Firmicutes and Bacteroidetes. Data are expressed as mean ± S.D., where different letters indicate statistical difference, *p* < 0.05.

Four main phyla were characterized—Firmicutes, Bacteroidetes, Actinobacteria, and Proteobacteria. There was no statistical difference between two lard groups LF and LO across phyla (*p* > 0.05). However, feeding a CO diet significantly reduced Proteobacteria and Bacteroidetes, whereas it increased Actinobacteria, compared with feeding CF (*p* < 0.05). The ratio of Firmicutes to Bacteroidetes was significantly higher in the CO group compared with CF (*p* < 0.05) (Figure 7).

### 3.11. Family Abundance

**Figure 8:**
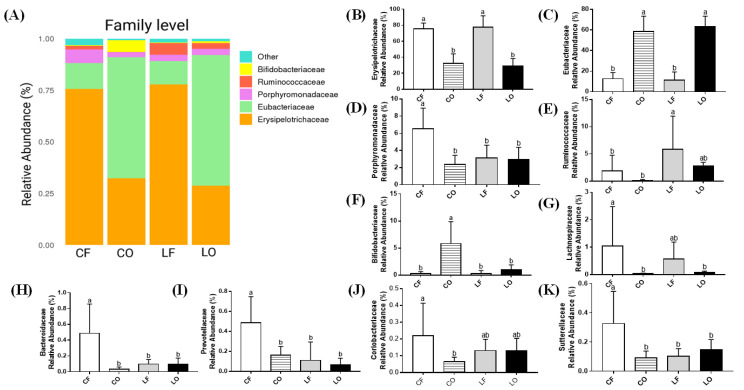
Family-level changes in bacterial composition of hamsters fed one of the four diets: CF, fresh corn oil; CO, oxidized corn oil; LF, fresh lard; LO, oxidized lard. (**A**) Bar plot of relative abundance (%). Comparison in abundances of: (**B**) Erysipelotrichaceae, (**C**) Eubacteriaceae, (**D**) Porphyromonadaceae, (**E**) Ruminococcaceae, (**F**) Bifidobacteriaceae, (**G**) Lachnospiraceae, (**H**) Bacteroidaceae, (**I**) Prevotellaceae, (**J**) Coriobacteriaceae, and (**K**) Sutterellaceae. Data are expressed as mean ± S.D., where different letters indicate statistical difference, *p* < 0.05.

Both oxidized groups, CO and LO, had significantly lower abundance of Erysipelotrichaceae, and higher Eubacteriaceae at the family level compared with CF and LF, respectively (*p* < 0.05). Lard groups LF and LO had comparable abundance of Porphyromonadaceae, Bifidobacteriaceae, Lachnospiraceae, Sutterellaceae, Coriobacteriaceae, Bacteroidaceae, and Prevotellaceae (*p* > 0.05). In the two corn oil groups, however, feeding a CO diet led to a major reduction in Porphyromonadaceae, Coriobacteriaceae, Lachnospiraceae, Prevotellaceae, Bacteroidaceae, and Sutterellaceae (*p* < 0.05). Feeding CO had elevated Bifidobacteriaceae compared with feeding CF (*p* < 0.05). The abundance of Ruminococcaeae remained unaffected by the intake of oxidized LO and CO, relative to LF and CF, respectively (*p* > 0.05) (Figure 8).

### 3.12. Genus Abundance

**Figure 9:**
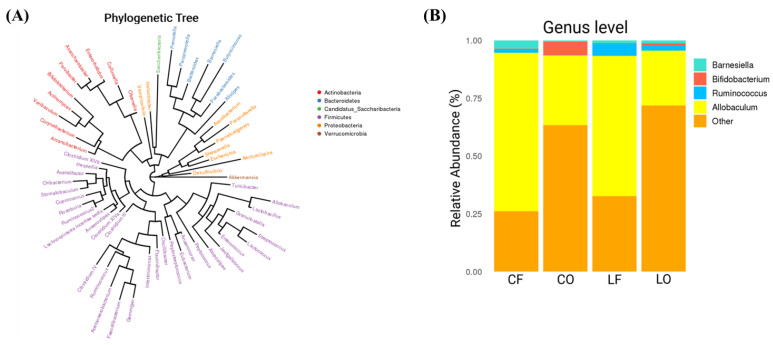
(**A**) Phylogenetic tree depicting the evolutionary relationship between the genera and phyla of bacteria found in this study. (**B**) Bar plot of genus relative abundance (%) between hamsters fed one of the four diets: CF, fresh corn oil; CO, oxidized corn oil; LF, fresh lard; LO, oxidized lard.

**Figure 10:**
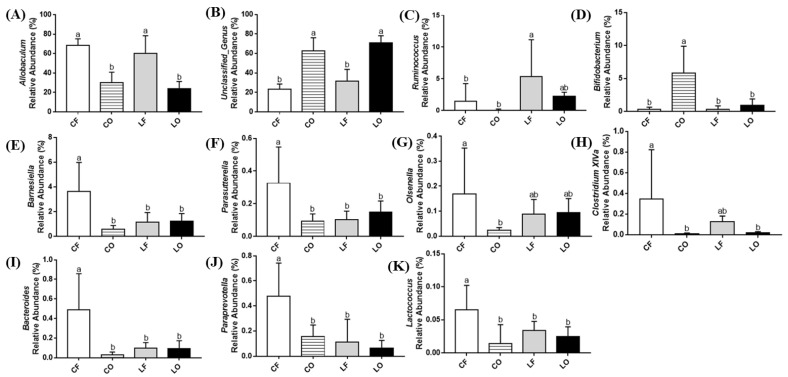
Genus level changes in bacterial composition of hamsters fed one of the four diets: CF, fresh corn oil; CO, oxidized corn oil; LF, fresh lard; LO, oxidized lard. Comparison of relative abundances of: (**A**) *Allobaculum*, (**B**) *Unclassified_Genus* (**C**) *Ruminococcus*, (**D**) *Bifidobacterium*, (**E**) *Barnesiella*, (**F**) *Parasutterella*, (**G**) *Olsenella*, (**H**) *Clostridium XIVa,* (**I**) *Bacteroides*, (**J**) *Paraprevotella*, and (**K**) *Lactococcus.* Data are expressed as mean ± S.D., where different letters indicate statistical difference, *p* < 0.05.

Differences in genus abundance among the four diet groups were also observed. The taxonomic relationship between genera and phyla is represented by the phylogenetic tree (Figure 9A). Compared with the fresh groups CF and LF, both oxidized groups CO and LO had significantly reduced the abundance of *Allobaculum* (*p* < 0.05). Both oxidized and fresh groups had similar levels of *Ruminococcus* (*p* > 0.05). Between the two lard groups, LF and LO did not have significant alterations in the abundances of *Bifidobacterium, Barnesiella, Parasutterella, Olsenella, Lactococcus, Paraprevotella, Clostridium_XIVa,* and *Bacteroides* (*p* > 0.05). However, in the corn oil groups, oxidized CO intake led to a higher abundance of *Bifidobacterium,* and a drastic reduction in *Barnesiella, Parasutterella, Olsenella, Lactococcus, Paraprevotella, Clostridium_XIVa,* and *Bacteroides* (*p* < 0.05) (Figure 10). Analysis of genus abundance found that a significant number of *Unclassified_Genus* was elevated in oxidized CO and LO, which were categorized as “Other” (*p* < 0.05) (Figure 9B) (Figure 10B).

### 3.13. Correlation of Genus Abundance with Metabolic Parameters

**Figure 11:**
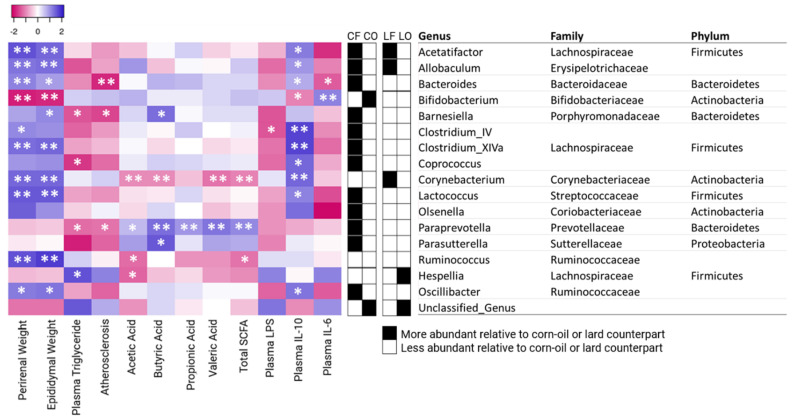
Spearman’s correlation heatmap of major genera with key indices. Hamsters were fed one of the four diets: CF, fresh corn oil; CO, oxidized corn oil; LF, fresh lard; LO, oxidized lard. Correlation significance is indicated by asterisks (“*” reflects *p* < 0.05, “**” *p* < 0.01). Filled box (▪) represents a corn oil or lard group that has a significantly greater abundance of bacteria relative to its counterpart (*p* < 0.05). Unfilled box (▫) represents a corn oil or lard group that has a significantly lower abundance of bacteria relative to its counterpart (*p* < 0.05). Taxonomic information (phylum, family) is provided on the right.

Spearman’s correlation analyses were performed to correlate the abundance of specific bacterial genus with other parameters, including weight of perirenal and epididymal adipose tissues, plasma TG, atherosclerotic plaque area, total and individual SCFAs (acetic acid, butyric acid, propionic acid, valeric acid), as well as plasma LPS, IL-10, and IL-6. The weight of perirenal and epididymal adipose tissue was found to have a direct correlation with *Acetatifactor*, *Allobaculum, Bacteroides, Clostridium_IV, Clostridium_XIVa, Corynebacterium, Lactococcus, Ruminococcus,* and *Oscillibacter*, while it indirectly correlated with *Bifidobacterium* (*p* < 0.05). Plasma TG had an inverse relationship with *Barnesiella, Coprococcus, and Paraprevotella*, and a direct association with *Hespellia* (*p* < 0.05). Levels of *Barnesiella, Bacteroides,* and *Paraprevotella* were inversely correlated with atherosclerotic plaque accumulation (*p* < 0.05). Total SCFA was indirectly linked with *Corynebacterium* and *Ruminococcus*, while it was directly connected to the abundance of *Paraprevotella* (*p* < 0.05). Plasma inflammatory IL-10 was indirectly correlated to *Bifidobacterium*, and had a direct association to *Acetatifactor, Allobaculum, Bacteroides, Clostridium_IV, Clostridium_XIVa, Coprococcus, Corynebacterium, Lactococcus,* and *Oscillibacter* (*p* < 0.05) (Figure 11).

## 4. Discussion

The expansion of the global fast-food industry has led to an increase in the consumption of deep-fried food products. Complex chemical reactions during deep-frying produce oxidative products, which are largely absorbed into the food [3]. It remains unclear how different vegetable oils and animal fats can affect the gut microbiota when they are used repeatedly in deep-frying. The present study aimed to investigate and compare the effect of thermally-oxidized corn oil (vegetable oil) and lard (animal fat) on gut microbiota. By mimicking common restaurant practices, the oxidized oil and lard were prepared according to traditional French fry recipes for seven consecutive days. Golden Syrian hamsters were fed diets containing oxidized and fresh corn oil and lard for six weeks. It was observed that the intake of thermally-oxidized corn oil (CO) and oxidized lard (LO) caused some significant changes in the overall structure and composition of gut bacteria. The results of alpha diversity index analysis clearly demonstrated that CO and LO caused a marked loss of species diversity and richness. The compositional changes across bacteria phylum, family, and genus levels were also shown to be associated with atherosclerotic plaque development, gut-derived SCFA, and inflammatory biomarkers LPS, IL-6, and IL-10.

As valid indicators of gastrointestinal health and functioning, alpha diversity indices (Sobs and Shannon) are used to effectively compare gut bacterial communities under various physiological condition [20]. The structure and diversity of gut microbiota can be measured in terms of the number of unique species (richness), evenness within a sample, and a combination of the two [21]. Compared with the fresh CF and LF groups, the oxidized CO and LO groups had significantly lower species richness and diversity (Figure 5). Sufficient sampling was conducted and indicated by the rarefaction curves of both the Sobs and Shannon index (Figure 5). Differences between community structure could also be seen through PCA analysis of OTUs, which showed a clear separation of CO from CF, and LO from LF, along the PC1 axis (Figure 6). Additionally, the number of unique OTUs was also lower in the oxidized CO and LO groups, compared with their non-oxidized counterparts CF and LF, as represented by Venn diagrams (Figure 4). The loss of alpha diversity has been linked to metabolic complications such as obesity, irritable bowel syndrome (IBD), diabetes, and metabolic syndrome [22,23,24,25].

A decrease in bacterial diversity and richness was found to significantly affect healthy body weight. Previous studies had observed that a key difference between abnormal weight (both over- and under-weight), adiposity, dyslipidemia, and elevated inflammation, is characterized by the loss of bacterial richness [26,27]. Similarly, the present study also observed that the body weights of CO were 15% lower than CF, while LO was 7% lower than LF, despite of similar food intakes among four groups (Table 3). This was accompanied by a 15–35% reduction of epididymal and perirenal adipose tissues in the oxidized groups CO and LO, compared with the fresh CF and LF groups (Table 3). Spearman’s correlation found that the genus abundance of *Acetatifactor* and *Allobaculum* was positively correlated with epididymal and perirenal fat weights (Figure 11). Feeding CO and LO diets caused a reduction in the abundance of both genera, consistent with the reduction in adipose tissue weights compared with CF and LF, respectively. In addition, CO also had a lower abundance of other genera which were positively correlated with epididymal and perirenal fat, including: *Bacteroides, Clostridium_XIVa, Lactococcus, Oscillibacter, Barnesiella,* and *Clostridium_IV* (Figure 10). Additionally, CO had elevated *Bifidobacterium*, which is negatively associated with both adipose tissue weights. It has been shown that *Bifidobacterium* is associated with the lean phenotype in other studies [28]. Decreased body weight and body fat content after the consumption of reused frying oils and lipid oxidation products have also been observed in porcine and canine models [29,30]. It has also been found that the oxidation products may decrease overall digestibility, due to the difficulty in polymer breakdown and absorption [31,32]. This could explain the elevated weight of weekly fecal output observed in the oxidized groups in our study, where the fecal weights of CO and LO were 93–111% higher than those of the CF and LF groups (Table 3). Interestingly, while the body weights of oxidized groups were reduced, the plasma TG levels of CO and LO were 35–50% higher than CF and LF (Table 4). It was observed that *Barnesiella*, which is associated with epididymal weight, was inversely correlated with plasma TG (Figure 11). It is speculated that the decrease of *Barnesiella, Coprococcus, and Paraprevotella* in the CO group, and the increase of *Hespellia* in the LO group could contribute to the increased TG levels, compared with CF and LF (Figure 10). Other studies have also found that gut microbiota could affect TG deposition into adipocytes via fasting-induced adipocyte factor [33], which may explain the differences in plasma TG, adipose tissues, and body weights between oxidized and non-oxidized groups of corn oil and lard. The present results suggested that the loss of bacterial richness and diversity in CO and LO was, at least in part, responsible for the reduction in body weight and adipose tissue. It is also worth noting that other oxidation products produced during frying process such as *trans* fatty acids, had been found to increase plasma TG levels similarly to our observation, although previous studies in this regard have been highly inconsistent [34,35,36,37].

Inflammation is regulated by the action of cytokines such as interleukin (IL)-6 and IL-10. Increased IL-6 levels promote chronic inflammation, which is characteristic in patients with inflammatory diseases like rheumatoid arthritis, autoimmune diseases, as well as in COVID-19 [38,39]. Contrastingly, the anti-inflammatory mediator IL-10 assists in regulating host immune response. Dysregulated IL-10 levels are associated with inflammatory complication and infections [40,41]. The measurement of plasma cytokines found that the oxidized CO group had significantly elevated IL-6 concentration and decreased levels of plasma IL-10, compared with the CF group, whereas no differences were observed between plasma cytokines of the lard LO and LF groups (Figure 2). Previous studies using probiotics have found that gut bacteria can influence plasma cytokines [42,43]. Correlation analysis found that plasma IL-6 was negatively correlated with *Bacteroides* genus and positively correlated with *Bifidobacterium* in our study (Figure 11). Reduced abundance of *Bacteroides* and elevated *Bifidobacterium* in CO may partly contribute to the elevated plasma IL-6 compared with CF (Figure 10). The release of inflammatory cytokines such as IL-6 can be induced by endotoxin LPS, which enters circulation through leaky gut barriers [44]. The relationship between LPS and IL-6 is supported in our study, as the data of plasma LPS compliments IL-6. The CO group had higher LPS concentration compared to CF, and no significant differences between two lard groups were observed (Figure 2). Compared to CF, CO had significantly lower *Clostridium_IV* abundance, which is a bacteria cluster negatively correlated with plasma LPS (Figure 11). Anti-inflammatory plasma IL-10 also had a direct correlation with several gut bacteria, specifically: *Acetatifactor, Allobaculum, Bacteroides, Clostridium_IV, Clostridium_XIVa, Coprococcus, Lactococcus*, and *Oscillibacter* (Figure 11). Compared with the CF group, the abundance of these genera in the CO group was significantly reduced, which could explain the reduction in the plasma IL-10 level of the CO group (Figure 2).

Atherosclerosis and the build-up of fatty streak and plaques are accelerated by aberrant levels of IL-6 and IL-10 [45,46]. The present study observed that the atherosclerosis plaque area of CO was higher than CF, with no difference being observed between LO and LF (Figure 1). This trend was complementary to plasma IL-6 and IL-10 levels, supporting the relationship between cytokines and atherosclerosis progression (Figure 2). Additionally, the percentage of plaque accumulation in the aorta was negatively linked to bacteria abundance of *Bacteroides, Barnesiella,* and *Paraprevotella* through Spearman’s correlation analysis (Figure 11). This suggests the role of gut bacteria in the promotion of aortic plaque development and atherosclerosis.

SCFAs are metabolites produced by gut bacteria that can regulate host metabolism. Studies have found the beneficial effects of SCFAs on immune regulation by suppressing production of pro-inflammatory cytokines such as IL-6, while increasing IL-10 secretion [47,48,49]. Total fecal SCFAs levels in CO were significantly reduced compared with CF, in a similar trend to plasma IL-6 and IL-10 data (Figure 2 and Figure 3). Similarly, an inverse relationship between fecal SCFAs and plasma LPS in the CO group was also observed (Figure 2 and Figure 3). Previous studies have found that SCFAs can promote a healthy gut barrier and prevent inflammation caused by the entrance of LPS into bloodstream [50]. As a gut-derived metabolite, SCFAs production can be attributed to multiple bacterial genera. *Paraprevotella* of the Bacteroidetes phylum was directly associated with the production of acetate, butyrate, propionate, valerate, and total fecal SCFAs. Feeding CO caused a significant decrease in *Paraprevotella* and Bacteroidetes phyla, compared with the CF group (Figure 10). Additionally, *Parasutterella* and *Barnesiella* (both directly linked to butyrate), were also decreased in the CO group. On a phylum level, Bacteroidetes phyla was significantly reduced by the intake of oxidized CO compared with CF, leading to a higher Firmicutes to Bacteroidetes (F:B) ratio. The F:B ratio is a debated indicator of metabolic health, body weight maintenance, and immune regulation [51,52,53]. An increased F:B ratio in the CO group could potentially explain the elevated inflammation and body weight differences relative to those in the CF group.

Fresh and oxidized lard and corn oil had different effects on gut microbiota. This may be attributable to differences in their fatty acid compositions (Table 2). Fresh lard (in LF) largely consisted of 45% SFA and 37% MUFA. Fresh corn oil (in CF), on the other hand, has 53% PUFA and 30% MUFA. With a higher level of unsaturation, corn oil is less stable than lard, and is more susceptible to oxidation and rancidity [54]. Repeated deep-frying led to a significant loss of PUFA in corn oil (CF), making MUFA and SFA proportionally the major fatty acids in CO (Table 2). On the other hand, while thermal-oxidation did increase the saturation degree of lard, both LO and LF remained mainly composed of SFA and MUFA. As a result, differences between the lard LO and LF groups in gut microbiota may not be as radical as the differences observed between the corn oil CO and CF groups.

There are several limitations in the study of thermally-oxidized deep-frying oil and health. Deep-frying is an intricate process and specific oxidation products produced have not been fully identified [55]. Furthermore, differences in experimental conditions (e.g., choice of oil and their fatty acid profiles, choice of food for frying, temperature of oil, and frequency of frying) can affect the degree of changes to oil quality, leading to different findings between studies. Similarly, much remains to be investigated about how reused deep-frying oils can affect specific bacteria in the gut, as our study also observed that oxidized CO and LO had significant elevation in *Unclassified_Genus* abundance, which are yet to be identified.

## 5. Conclusions

In summary, the current study aimed to investigate how the intake of repeatedly reused deep-frying corn oil and lard could affect gut microbiota, compared to the consumption of fresh oil and lard. It was observed that oxidized oil and fat could decrease the richness and diversity of gut composition in hamsters. Corn oil, in particular, was more susceptible to changes as a result of repeated deep-frying and could alter gut microbiota at multiple taxonomic levels. The loss of bacterial richness and diversity, and the alteration of specific bacterial abundances, could exacerbate the atherosclerotic plaque development, and dysregulate the production of gut-derived metabolite SCFAs, pro-inflammatory biomarkers LPS and IL-6, and anti-inflammatory biomarker IL-10.

## Figures and Tables

**Table 1:** Composition of the four diets: CF, fresh corn oil; CO, oxidized corn oil; LF, fresh lard; LO, oxidized lard.

Ingredients (g/kg)	CF	CO	LF	LO
Corn Starch	508.0	508.0	508.0	508.0
Casein	242.0	242.0	242.0	242.0
Sucrose	119.0	119.0	119.0	119.0
Corn Oil—Fresh	155.0	-	-	-
Corn Oil—Oxidized	-	155.0	-	-
Lard—Fresh	-	-	155.0	-
Lard—Oxidized	-	-	-	155.0
Mineral Mix	40.0	40.0	40.0	40.0
Vitamin Mix	20.0	20.0	20.0	20.0
Gelatin	20.0	20.0	20.0	20.0
DL-Methionine	1.0	1.0	1.0	1.0
Cholesterol (0.04%)	0.4	0.4	0.4	0.4

**Table 2:** Fatty acid composition and peroxide values of fresh corn oil (CF), oxidized corn oil (CO), fresh lard (LF), and oxidized lard (CO).

	CF	CO	LF	LO
***(a) Fatty Acids (%)***				
**14:0**	1.68	3.06	1.75	3.20
**16:0**	11.48	17.02	25.86	35.97
**18:0**	1.65	2.56	16.90	18.87
***Total SFA***	14.82	22.65	44.52	58.05
**18:1n-9**	29.98	38.11	37.46	27.31
***Total MUFA***	29.98	38.11	37.46	27.31
**18:2n-6**	51.76	33.36	9.98	0.71
**18:3n-3**	0.83	0.23	0.75	0.42
***Total PUFA***	52.60	33.59	10.74	1.14
***(b) Peroxide value (mEq peroxide/kg sample)***	5.34 ± 0.22 ^c^	33.15 ± 1.91 ^a^	2.65 ± 0.08 ^c^	12.51 ± 0.55 ^b^

SFA, saturated fatty acids; MUFA, monounsaturated fatty acids; PUFA, polyunsaturated fatty acids. Different letters in superscript indicate statistical difference, *p* < 0.05.

**Table 3:** Food intake, body weight, organ weight, and fecal output of hamsters fed one of the four diets: CF, fresh corn oil; CO, oxidized corn oil; LF, fresh lard; LO, oxidized lard. *

	CF	CO	LF	LO	*p*-Value
***Daily Food Intake (g)***	7.83 ± 0.59	7.30 ± 0.36	7.25 ± 0.48	7.79 ± 0.21	0.137
***Body Weight (g)***					
Initial	107.88 ± 6.38	109.00 ± 6.57	108.88 ± 7.20	106.75 ± 9.51	0.926
Final	137.00 ± 8.04 ^a^	115.88 ± 8.13 ^c^	135.57 ± 7.04 ^a^	125.71 ± 7.50 ^b^	<0.001
***Organ Weights (g/100 g Body Weight)***					
Heart	0.80 ± 0.03 ^b^	0.90 ± 0.05 ^a^	0.81 ± 0.05 ^b^	0.84 ± 0.04 ^b^	<0.001
Testis	3.61 ± 0.25 ^b^	4.06 ± 0.30 ^a^	3.76 ± 0.41 ^ab^	3.67 ± 0.19 ^b^	0.025
Kidney	1.30 ± 0.06 ^ab^	1.51 ± 0.08 ^c^	1.28 ± 0.05 ^a^	1.43 ± 0.06 ^b^	<0.001
Liver	5.33 ± 0.22 ^c^	6.06 ± 0.34 ^a^	5.18 ± 0.24 ^c^	5.66 ± 0.26 ^b^	<0.001
Perirenal Adipose Tissue	1.70 ± 0.31^ab^	1.31 ± 0.11^c^	1.81 ± 0.12 ^a^	1.55 ± 0.08 ^bc^	<0.001
Epididymal Adipose Tissue	2.25 ± 0.38 ^c^	1.88 ± 0.18 ^a^	2.45 ± 0.27 ^c^	2.02 ± 0.28 ^b^	0.002
***Weekly Fecal Output (g*** *)*	3.58 ± 0.61 ^b^	7.57 ± 0.97 ^a^	3.68 ± 0.64 ^b^	7.10 ± 0.63 ^a^	<0.001

* Data are expressed as mean ± S.D., where different letters in superscript indicate statistical difference, *p* < 0.05.

**Table 4:** Plasma lipid profile at Week 0 and Week 6 of hamsters fed one of the four diets: CF, fresh corn oil; CO, oxidized corn oil; LF, fresh lard; LO, oxidized lard *.

	CF	CO	LF	LO	*p*-Value
***Week 0***					
**TC (mg/dL)**	144.96 ± 15.06	147.53 ± 13.48	144.66 ± 18.86	144.88 ± 23.96	0.988
**HDL-C (mg/dL)**	114.15 ± 5.84	115.40 ± 12.78	117.01 ± 5.94	115.25 ± 7.35	0.925
**nHDL-C (mg/dL)**	30.81 ± 17.73	32.13 ± 16.82	27.65 ± 19.95	29.63 ± 17.29	0.965
**nHDL-C/HDL-C**	0.27 ± 0.17	0.29 ± 0.18	0.24 ± 0.17	0.25 ± 0.14	0.919
**HDL-C/TC**	0.80 ± 0.10	0.79 ± 0.11	0.82 ± 0.12	0.81 ± 0.11	0.922
**TG (mg/dL)**	91.25 ± 21.30	96.50 ± 43.56	102.42 ± 23.68	98.25 ± 21.52	0.894
***Week 6***					
**TC (mg/dL)**	150.06 ± 15.32 ^bc^	137.56 ± 15.08 ^c^	189.18 ± 10.40 ^a^	164.43 ± 16.97 ^b^	<0.001
**HDL-C (mg/dL)**	133.88 ± 15.68 ^b^	134.18 ± 10.21 ^b^	156.16 ± 16.38 ^a^	145.10 ± 11.63 ^ab^	0.009
**nHDL-C (mg/dL)**	16.18 ± 12.99 ^a^	3.38 ± 7.54 ^c^	33.01 ± 14.81 ^b^	19.33 ± 11.69 ^a^	0.001
**nHDL-C/HDL-C**	0.13 ± 0.12 ^a^	0.02 ± 0.06 ^b^	0.22 ± 0.10 ^a^	0.13 ± 0.08 ^a^	0.003
**HDL-C/TC**	0.89 ± 0.08 ^b^	0.98 ± 0.06 ^a^	0.83 ± 0.08 ^b^	0.89 ± 0.06 ^b^	0.002
**TG (mg/dL)**	75.50 ± 25.45 ^c^	114.25 ± 30.82 ^b^	111.94 ± 21.13 ^b^	150.69 ± 30.84 ^a^	<0.001

* Data are expressed as mean ± S.D., where different letters in superscript indicate statistical difference, *p* < 0.05.

## Data Availability

All data produced in this study were presented in the article.
